# Implementing Sensitivity and Contingency in Medical Contexts: The Case of Prematurity

**DOI:** 10.3390/jcm12165306

**Published:** 2023-08-15

**Authors:** Silvia Cimino

**Affiliations:** Department of Dynamic, Clinical and Health Psychology, Sapienza University of Rome, 00185 Rome, Italy; silvia.cimino@uniroma1.it

In the context of relational situations, sensitivity and contingency are identified as pivotal variables that contribute to the enhancement of patients’ overall wellbeing. Both physical and mental health are deeply affected by these factors. Caregivers, which encompass not only family members but also professionals dealing with situations of illness and distress, play a crucial role in fostering patients’ recovery [1,2].

The affective and relational skills of caregivers play a significant role in this process. When caregivers exhibit high levels of sensitivity, they are attuned to the emotional and psychological needs of their patients. They respond empathetically, providing comfort and understanding. On the other hand, contingency refers to the ability of caregivers to respond adaptively to the changing needs of patients. This responsiveness establishes a sense of security, predictability, and trust in the relationship [3].

Research suggests that the presence of sensitive and contingent caregiving can lead to accelerated recovery on both physical and mental fronts. Patients who receive such care are more likely to experience reduced stress, anxiety, and depression. Moreover, their physical healing processes may be expedited, potentially leading to shorter hospital stays and improved medical outcomes in different realms of physical conditions such as oncology, traumatic injuries, and cardiac problems [4,5].

This is particularly evident in cases of premature births, which have seen an increase in recent years due to various factors such as lifestyle changes, medical advancements, and environmental influences. Premature births are a notable area of study where the concepts of sensitivity and contingency have gained particular significance. Infants born prematurely often require specialized medical attention and care due to their underdeveloped physiological systems. In this context, the emotional and relational support provided by caregivers becomes even more critical. Sensitivity and contingency in caregiving can have a direct impact on the infant’s developmental trajectory, potentially mitigating the risks associated with preterm birth [6,7].

In general, the constructs of sensitivity and contingency hold a central place in the scientific exploration of psychology, developmental psychopathology, and clinical medicine. All these standpoints underscore the importance of nurturing responsive, empathetic, and adaptable caregiving relationships to enhance the wellbeing of patients across various stages of life [8,9,10,11].

Particularly in the case of premature births, these constructs can significantly influence the developmental outcomes of infants facing unique challenges.

Premature births, defined as births occurring before the completion of 37 weeks of gestation, have been identified as a significant contributor to various neurodevelopmental difficulties. These challenges can have lasting effects throughout an individual’s life cycle. From the early stages of infancy through later life, individuals born prematurely often require a unique and carefully tailored relational environment that is characterized by sensitivity and attentiveness to their needs.

Neurodevelopmental difficulties associated with premature birth can encompass a range of issues, including cognitive impairments, motor delays, behavioral challenges, and emotional regulation difficulties. These difficulties may persist into adulthood and impact an individual’s education, employment, and overall quality of life [12].

Given the distinct vulnerabilities that premature infants face, it becomes crucial to provide a relational environment that is attuned to their specific needs. In this context, caregiver sensitivity plays a pivotal role. Caregiver sensitivity refers to the ability of parents, caregivers, and healthcare professionals to accurately perceive and appropriately respond to the cues and needs of the infant, even when these cues might not be as overt as in full-term infants.

Research in this area has explored various techniques aimed at promoting caregiver sensitivity towards preterm infants. These techniques are particularly important because preterm infants might not yet have developed the ability to clearly manifest their needs and requirements. Psychodynamically oriented pre-term parenting support programs, for example, focus on enhancing parents’ and caregivers’ awareness and responsiveness to the unique needs of premature infants [13,14]. These programs often involve teaching caregivers how to interpret subtle cues, providing guidance on how to regulate their emotions, and facilitating a deeper understanding of the infant’s experiences.

The hospital setting, where premature infants often receive initial care, is a crucial context for the implementation of caregiver sensitivity techniques. Training caregivers in this environment is essential to ensure that they are equipped with the skills necessary to provide the best possible care for premature infants. Caregivers need support in expressing their own emotions, distinguishing their personal experiences from those of the premature baby, and managing any projections they might have onto the infant [15].

For instance, caregivers might experience feelings of guilt or inadequacy related to premature birth, which could hinder the formation of a healthy attachment bond. They might struggle to accept the reality of the situation, as it might differ significantly from their expectations of a “typical” birth experience. These complex emotions and projections can potentially impact the caregiver–infant relationship, underscoring the importance of addressing caregivers’ emotional needs as part of the support process [16].

In conclusion, premature births have been linked to neurodevelopmental difficulties that can span an individual’s life cycle. Creating a sensitive and attentive relational environment is essential for addressing the unique needs of these individuals. Techniques aimed at enhancing caregiver sensitivity, especially in psychodynamically oriented pre-term parenting support programs, can play a crucial role in improving outcomes for premature infants. Training caregivers, particularly within the hospital context, is vital for equipping them with the skills to navigate the complex emotions and challenges associated with premature birth and its impact on the caregiver–infant relationship [17].

Among the clinical approaches that foster the development of caregiver sensitivity and contingency, home visiting interventions are particularly interesting [18,19,20]. These are programs for the prevention of psycho-physical distress, which accompany families from the first months of a child’s life. Although there are still few experiences in Italy, in the Anglo-Saxon and American worlds, clinical approaches are widely used at the time of a patient’s discharge. It is a question of offering home treatment that allows the people involved in care and nursing to find new and more appropriate ways of communicating in the home context, which favor two-way communication, mutual emotional contact, and emerging states of affective synchronization.
